# Serratia Marcescens, a Rare and Devastating Cause of Endocarditis: A Case Report and Review of the Literature

**DOI:** 10.7759/cureus.25572

**Published:** 2022-06-01

**Authors:** Kelly Luttmann, Victoria R Starnes, Michael Haddad, Joan Duggan

**Affiliations:** 1 Infectious Disease, University of Toledo Medical Center, Toledo, USA; 2 Infectious Disease, University of Toledo, Toledo, USA

**Keywords:** serratia endocarditis, endocarditis, opportunistic infection, serratia marcescens endocarditis, serratia marcescens, infective endocarditis

## Abstract

*Serratia marcescens* is a gram-negative bacillus that is an opportunistic agent in respiratory tract infections, urinary tract infections, and septicemia. It is rarely a cause of infective endocarditis, but in cases of endocarditis, it follows a rapid and devastating course.

A previously healthy female in her mid-50s presented with fever, abdominal pain, right lower extremity pain, and diarrhea. Blood cultures were positive for *S. marcescens,* and additional evaluation revealed infarction in the spleen and kidneys, raising concern for endocarditis with associated embolic phenomena. The patient was subsequently found to have an embolus in the right popliteal artery and underwent a right popliteal thromboembolectomy. Antimicrobial therapy with cefepime and gentamicin was begun. A transesophageal echocardiogram revealed a large, mobile mitral valve vegetation. Care was complicated by intracranial hemorrhage, and the decision was made to withdraw care.

A review of the databases Embase and PubMed revealed 63 additional cases of *S. marcescens* endocarditis. Analysis of these cases demonstrated a preponderance of aortic and mitral valve involvement, not tricuspid valve involvement, despite a risk factor of intravenous drug use in over 60% of cases. Mortality was 50%, and sequelae such as congestive heart failure and renal insufficiency occurred in the majority of survivors. In conclusion, *S. marcescens* is a rare but devastating cause of endocarditis with a primary risk factor of intravenous drug use but with a predilection for left-sided valvular lesions, not right-sided lesions.

## Introduction

*Serratia marcescens* is a motile, non-endospore-forming gram-negative bacillus [1]. This pathogen is an oxidase-negative, DNase-producing bacterium that is capable of growing in extreme conditions. Some strains of *S. marcescens* produce prodigiosin that results in a red pigment; these are known as pigmented S. marcescens. In contrast, nonpigmented *S. marcescens* lack this defining feature. Endocarditis due to *S. marcescens* was first described in 1951 secondary to pigmented strains and in 1970 from nonpigmented strains [2,3]. *S. marcescens* is commonly noted to be an opportunistic pathogen and is often associated with nosocomial infection. It can be an etiological agent in respiratory tract infections, urinary tract infections, septicemia, meningitis, and wound infections but rarely infective endocarditis. Due to increasing antibiotic resistance, *S. marcescens* may be difficult to treat.

While Serratia species are a rare cause of endocarditis, it often follows a rapidly progressive and devastating course. A major risk factor that has recently been reported with *S. marcescens* infective endocarditis is people who inject drugs (PWID) [2]. In a recent review of 43 cases of non-HACEK (*Haemophilus species*,* Aggregatibacter actinomycetemcomitans*,* Cardiobacterium hominis*,​​​​​​​* Eikenella corrodens*, and *Kingella kingae*) gram-negative rod infective endocarditis, nine cases of *S. marcescens* infective endocarditis were reported, and antimicrobial resistance was seen in 0/9 cases. The majority of cases in the series were associated with PWID (40/43 cases: 93%) and were right-sided lesions (27/43 cases: 63%), but this data was reported in aggregate, and no additional information specific to *S. marcescens* infective endocarditis was reported. All patients in this review received active empiric and definitive antimicrobial therapy with one or two agents. An overall mortality rate of 30% (13/43) at 12 months follow-up was reported, and left-sided infective endocarditis (IE) was independently associated with increased mortality [4]. We present a case of *S. marcescens* mitral valve endocarditis in a patient with a reported risk factor of PWID and a review of 63 additional cases in the English-language literature, discussing the risk factors, signs, symptoms, treatment, and outcomes.

## Case presentation

A previously healthy female in her 50s presented to a regional hospital with a one-week history of fevers, abdominal pain, and diarrhea as well as right lower extremity pain. Initial workup revealed an embolus in the right popliteal artery as well as lesions consistent with infarction in the spleen and bilateral kidneys on abdominal CT scan. The patient underwent urgent thromboembolectomy of the right popliteal artery, and blood cultures taken on admission were initially reported as positive for gram-negative rods. Meropenem 500 mg intravenously every six hours was started empirically while awaiting final identification of the bacteria and antibiotic sensitivities. The blood cultures were identified as *S. marcescens*, and antibiotic sensitivities revealed resistance to ampicillin, ampicillin/sulbactam, and cefazolin but sensitivity to fluoroquinolones, aminoglycosides, and third-generation cephalosporins.

**Figure 1:**
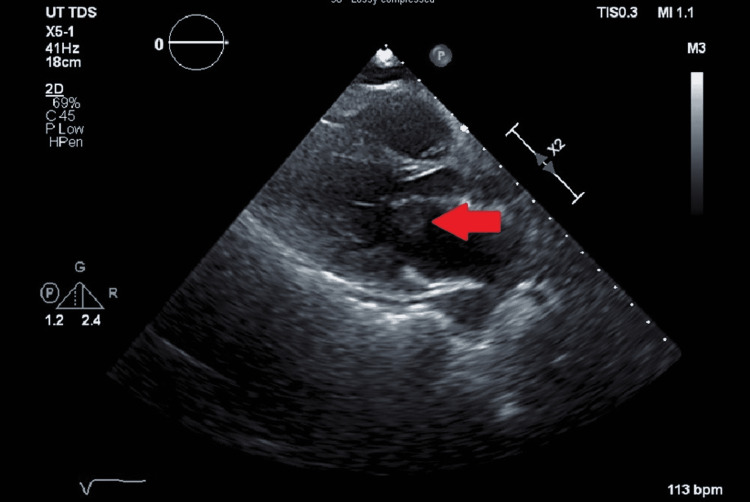
Two-dimensional transthoracic echocardiogram parasternal long-axis view, demonstrating the vegetation on the atrial aspect of the anterior mitral leaflet

Definitive therapy with cefepime 2 g intravenously every eight hours and gentamicin 240 mg intravenously daily was then initiated, and meropenem was discontinued. The findings of bacteremia with right popliteal artery occlusion raised concern for endocarditis with embolic phenomena. A transesophageal echocardiogram was performed and revealed a large, mobile mitral valve vegetation on the anterior leaflet measuring 2.8 cm x 1.5 cm (Figure 1). Serial blood cultures were obtained, and the bacteremia was noted to have cleared within four days of hospitalization, but despite this, the patient had persistent leukocytosis and remained febrile.

**Figure 2:**
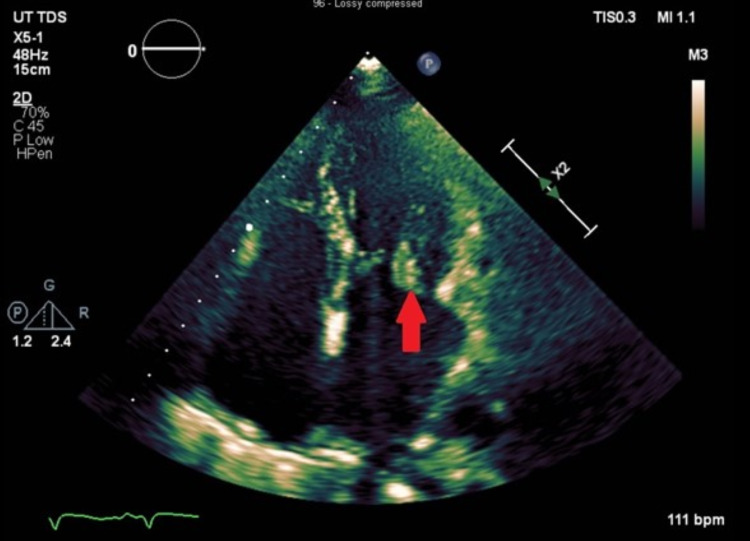
Mitral valve vegetation on the posterior leaflet seen in the apical four-chamber view transthoracic echocardiogram

**Figure 3:**
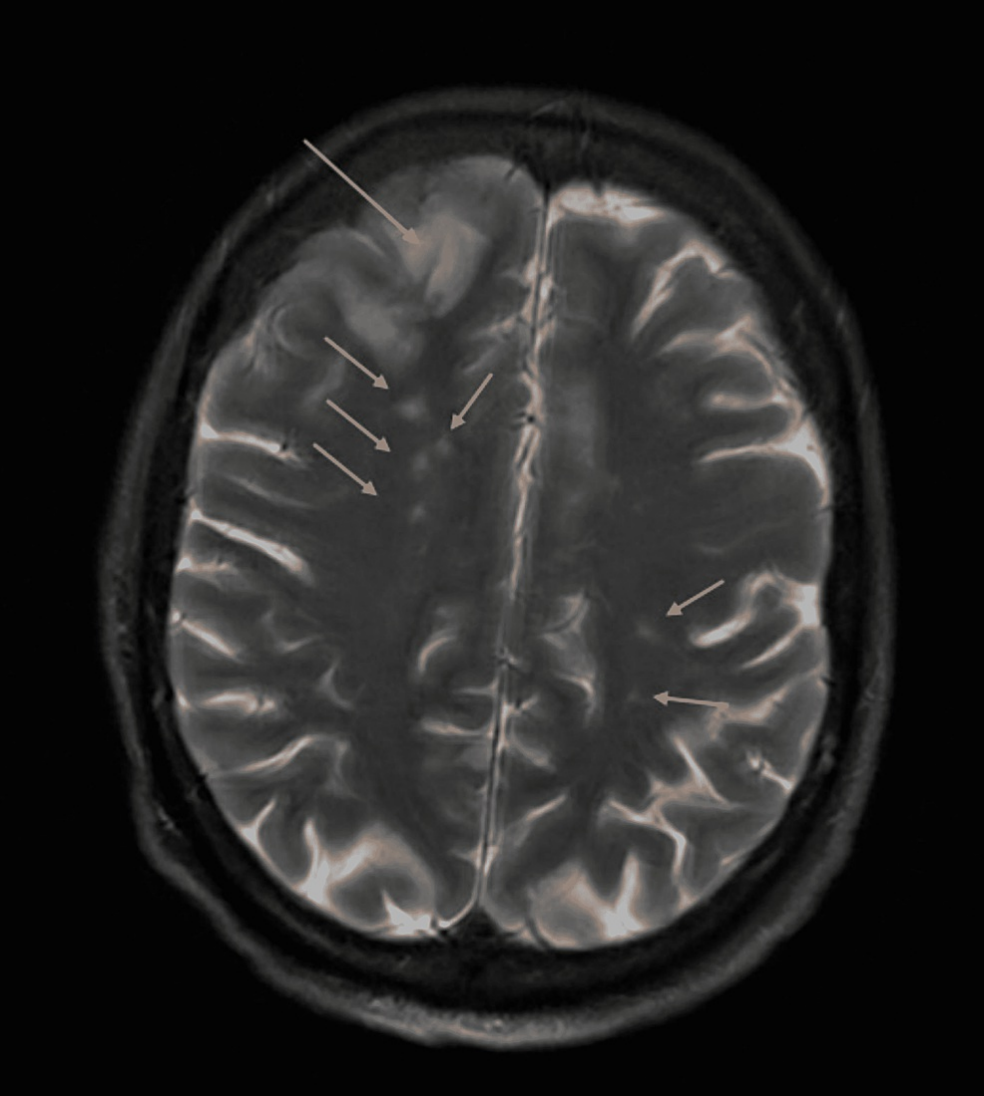
T2 MRI of the brain demonstrating multiple punctate foci in the bilateral frontal and parietal lobes consistent with embolic infarcts as well as subarachnoid hemorrhage in the right frontal lobe

The patient denied any risk factors for infective endocarditis such as recent dental work, recent intravenous lines, recent surgical procedures, or use of intravenous drugs or substance abuse. A family member stated that the patient had a history of substance abuse and PWID, but this was verified by the patient. Over the next few days, the patient progressively became more confused and somnolent, and an MRI of the brain revealed multiple punctate foci in the bilateral frontal and parietal lobes consistent with embolic infarcts as well as subarachnoid hemorrhage in the right frontal lobe (Figures 2, 3).

Due to these findings, cardiothoracic surgery was unable to perform mitral valve repair as the patient would be unable to tolerate anticoagulation following the procedure. One week after presentation, the patient developed tachypnea and hypoxia secondary to acute heart failure. The ejection fraction was estimated to be 55%, but moderate mitral valve regurgitation was noted on the transesophageal echocardiogram (TEE). After diuresis and stabilization of her clinical status, the decision was made to transfer the patient to a tertiary referral center outside of the region for a possible debulking procedure to remove the vegetation. On her third day of hospitalization at the tertiary referral center, she became increasingly obtunded prompting intubation for airway protection. A CT scan of the head revealed new intracranial hemorrhages in multiple areas including the brain stem with significant mass effect. The decision was made to withdraw care, and the patient passed away shortly after.

## Discussion

Methods

Case reports of *S. marcescens* endocarditis were identified through a computer-generated search using Embase and PubMed. Search terms included “Serratia,” “endocarditis,” and “bacteremia.” Only cases from the English-language literature were used. Cases were included in the analysis if (1) there was sufficient demographic information available to allow identification of an individual patient and (2) the patient met the modified Duke criteria for infective endocarditis due to *S. marcescens* including the presence of *S. marcescens* on valvular specimens pre- or post-mortem.

Results

Demographics

A total of 63 cases of *S. marcescens* endocarditis in addition to the current case (64 cases total) were reported between 1964 and 2020 [3-41]. Patients' ages ranged from neonate (<4 weeks of age) to 85 years old with a mean age of 42.7 ± 19.5. These included 28 (43.8%) females and 35 (54.7%) males.

Comorbidities

Fifty-three (82.8%) of the presented cases reported underlying conditions and comorbidities that acted as risk factors (Table 1). The most common risk factor was PWID. Seventeen patients presented with more than one comorbidity.

**Table 1:** Comorbidities in 53 cases of Serratia marcescens endocarditis *Some patients had more than one underlying condition. **Coronary artery disease, aortic stenosis, patent foramen ovale, atrioventricular block, hypertension, and ventral septal defects. ***Pyelonephritis, chronic kidney disease, and renal failure. ^†^Nosocomial infection, cirrhosis, SLE, obesity, hepatitis, non-Hodgkin's lymphoma, dental work, HIV, HBV, and HCV. SLE: Systemic lupus erythematosus; HBV: Hepatitis B virus; HCV: Hepatitis C virus; HIV: Human immunodeficiency virus; PWID: People who inject drugs.

Condition or risk-associated status	No. (%) of patients*
PWID	32 (60.4)
Cardiac	18 (40.0)
Rheumatic heart disease	6 (11.3)
Congestive heart failure	3 (5.7)
Other**	11 (20.8)
Renal***	3 (5.7)
Diabetes mellitus	6 (11.3)
Other^†^	15 (28.3)

Diagnostic Findings

All but one case had positive blood culture data for at least one strain of *S. marcescens*. The case that did not have positive blood culture data was found to have vegetations secondary to *S. marcescens* on a prosthetic heart valve at autopsy [22]. Nine cases had multiple strains of Serratia on culture lab data, and nine cases had additional findings from blood cultures, including *Pseudomonas aeruginosa*, *Candida albicans*, and *Staphylococcus aureus*.

Echocardiogram findings were reported for 17 of the 64 (26.6%) cases in the literature. The location of vegetation was described in 46 of the 64 (71.9%) cases with the left-sided valves being more commonly affected. The mitral valve was the most common source of vegetations (23 cases), followed by the aortic valve [16], tricuspid valve [15], and pulmonic valve [1]. Vegetations were present on multiple valves in six cases. Table 2 describes the outcomes based on the affected valve.

**Table 2:** Outcomes based on the affected valve in 46 cases of Serratia marcescens endocarditis

Source of vegetations	Outcomes
Mitral (23)	Survived: 5 (21.7%)
	Died: 18 (78.3%)
Tricuspid (15)	Survived: 10 (66.7%)
	Died: 5 (33.3%)
Aortic (16)	Survived: 7 (43.8%)
	Died: 9 (56.3%)
Pulmonic (1)	Survived: 1 (100%)
	Died: 0

Signs and Symptoms

Thirty-eight cases involved documented signs and symptoms at initial presentation or during the patient’s hospital stay. Symptoms varied from constitutional symptoms (fever, cough, and night sweats) to new murmur or onset of arrhythmia (Table 3). The most frequently documented symptom was fever (73.7%). Ten cases presented with symptoms following a surgical procedure.

**Table 3:** Signs and symptoms in 38 cases of Serratia marcescens endocarditis *Some patients presented with more than one symptom. GI: Gastrointestinal.

Signs/Symptoms	No. (%) of patients*
Constitutional	
Fever	28 (73.7%)
Cough	3 (7.9%)
Night sweats	1 (2.6%)
Fatigue	6 (15.8%)
Chest discomfort/pain	3 (7.9%)
Murmur	8 (21.1%)
Arrhythmia	3 (7.9%)
Confusion	3 (7.9%)
Splenomegaly	5 (13.2%)
GI (diarrhea and abdominal pain)	5 (13.2%)

Treatment

For 20 of 64 cases, no information on antimicrobial therapy was presented. Eight underwent a surgical procedure including excision of the infected valve or removal of the prosthetic valve. Twenty-four patients received a combination antibiotic therapy with a beta-lactam or carbapenem plus an aminoglycoside. Ten patients received a cephalosporin plus gentamicin, and seven patients received a carbapenem plus an aminoglycoside. In surviving patients, the length of treatment ranged from two weeks to 18 weeks, with an average of 6.5 weeks ± 4.0. Twelve patients received mono-antibiotic treatment with five patients dying before completion of the regimen. Susceptibility data were provided in only two cases.

Complications and Outcomes

Outcomes were not provided in five cases. Complications included metastatic abscesses (15.3%), emboli (27.1%), and sepsis. In patients with metastatic abscesses, these were found in the spleen, kidneys, brain, interventricular septum, eye, liver, superior mesenteric artery, and iliac artery. Emboli were found in the brain, lung, eye, and lower extremities (femoral artery, iliac artery, popliteal artery).

Fourteen patients developed heart failure during their hospital course, with 13 of these patients dying (92.9%). Other reported complications were loss of vision (one patient) and decline in renal function (four patients). Mortality was 50% (32 patients), and causes of death included uncontrolled sepsis, heart failure, embolus, complications of stroke, myocardial infarction, and uncontrollable bleeding.

Discussion

*S. marcescens* is a member of the *Enterobacteriaceae *family and is often associated with significant antibiotic resistance. The frequency of *S. marcescens* infections has been increasing since 1960, and *S. marcescens* has been implicated as a cause of many different forms of infection ranging from sepsis to skin/soft tissue infections [1]. Infective endocarditis is a rare manifestation of *S. marcescens* infection, and when it occurs, it is typically associated with poor outcomes [2].

In this review, *S. marcescens* endocarditis was associated with PWID occurring on left-sided valves with high morbidity and mortality [41]. While mitral and aortic valve endocarditis can occur in PWID, the infecting organism is often a gram-positive coccus. In a recent review of left-sided endocarditis compared to right-sided endocarditis in PWID, gram-negative aerobic bacilli were found in only 4% of cases of left-sided endocarditis (two cases total) and 5% of cases of right-sided endocarditis (four cases total) [42]. The etiology of this phenomenon remains unknown, and there is no evidence that gram-negative rods such as *S. marcescens* have an increased ability to increase to the matrix ligands that are associated with sterile vegetation but are important in the etiology, perhaps additional factors such as increased inoculum dose may be important in the etiology of left-sided gram-negative infective endocarditis in PWID.

Since endocarditis requires both bacteremia and endothelial damage, PWID are typically more likely to have right-sided endocarditis due to injected particles damaging the valves as they travel from the peripheral vascular system to the heart. These particles are also likely to get trapped in the lungs, thereby preventing them from damaging the left-sided valves [43]. When left-sided endocarditis occurs, it is predominately associated with gram-positive organisms [42]. In this case series, *S. marcescens* was found to predominately infect left-sided heart valves even in PWID, though the mechanism remains unknown.

*S. marcescens* infections are often difficult to treat due to the presence of inducible beta-lactamases (IBL) and extended-spectrum beta-lactamases (ESBL), and there is no consensus on the treatment of these infections in clinical situations with potentially reduced bioavailability such as respiratory infections, bacteremia, deep wound infection, infective endocarditis, or in an immunocompromised host [44,45]. Tigecycline, a minocycline derivative, remains a theoretical option for multidrug-resistant enterobacteria as it retains its activity against ESBL and AmpC-producing bacteria [42].

In clinical practice, the most common treatment for ESBL-resistant organisms is carbapenems; however, if an ESBL-producing organism is not detected, antimicrobial therapy can be de-escalated to fourth-generation cephalosporins or piperacillin/tazobactam [46]. The role of aminoglycosides in the treatment remains unknown, and few studies have reported susceptibility data. Infective endocarditis with *S. marcescens *can frequently lead to valve destruction, paravalvular complications, and embolisms in addition to valvular vegetations [45]. Due to this, early surgical intervention is recommended in addition to susceptibility-guided antibiotic therapy in order to manage both the infection and the sequelae of valve leaflet and paravalvular tissue damage [44]. Unfortunately, morbidity and mortality from this infection remain high. Finally, this case series is limited by its retrospective nature, but it serves to draw attention to the fulminant disease course associated with infection with this pathogen.

## Conclusions

*S. marcescens* endocarditis is usually seen in PWID but is associated predominately with aortic and mitral valve involvement with emboli and valve dysfunction, not tricuspid valve involvement. Morbidity is high in this condition and attributable to complications such as congestive heart failure, stroke, and bleeding: Mortality in this review was 50%. Further study in this area is needed as optimal recommendations for antibiotic therapy and surgical management remain unknown.
